# Performance of body mass index and body fat percentage in predicting metabolic syndrome risk factors in diabetic patients of Yazd, Iran

**DOI:** 10.1186/s12902-022-01125-0

**Published:** 2022-08-31

**Authors:** Marzieh Shukohifar, Zohre Mozafari, Masoud Rahmanian, Masoud Mirzaei

**Affiliations:** 1 Diabetes Research Center, Shahid Sadoughi University of Medical Sciences, Yazd, Iran; 2 Yazd Cardiovascular Research Centre, Non-communicable Disease Research Institute, Shahid Sadoughi University, Yazd, Iran

**Keywords:** Obesity, Body mass index, Body fat percentage, Metabolic syndrome, Risk factor

## Abstract

**Background:**

Body Fat percentage (BFP) and body mass index (BMI) are used to measure obesity-related metabolic syndrome risk. The present study aimed to determine the values of percent body Fat and body mass index for predicting metabolic syndrome risk factors in diabetic patients of Yazd, Iran.

**Methods:**

A total of 1022 (499 males and 523 females) diabetic patients participated in this study. According to Asian BMI criteria, Overweight was diagnosed if a participant had a BMI ≥25 kg/m^2^ (both male and female) or BFP ≥25% for male and ≥ 32% for female. Based on calculated BMI and BFP and after adjusting for age, height, weight and smoking habits, the participants were classified into group A (normal weight and Non-Fat), group B (overweight and Non-Fat), group C (normal weight and Fat), and group D (overweight and Fat).

**Results:**

According to the results, the BMI of 23.4% were normal and BMI of 76.6% were overweight, respectively. Moreover, the BFP of 25.7 and 74.3% of the studied population were considered as Non-Fat and Fat, respectively. A strong relationship was found with respect to sex stratification; R^2^ = 0.79. For men, BMI can be a better predictor of hypertension and hypertriglyceridemia than BFP. For women, BMI was a better predictor of hyperglycemia than BFP. Moreover, BFP can be regarded as a better predictor of hyperglycemia in male group, while it was a good predictor of hypertension and hypertriglyceridemia and hypo HDL than BMI, in female group.

**Conclusion:**

Significant differences were observed between BMI and BFP to predict metabolic syndrome risk factors in diabetic patients across different sexes in our study population. In conclusion, both BMI and BFP should be considered in screening steps.

## Background

Body mass index (BMI) is widely used in the diagnosis of overweight and obesity, whereas waist circumference (WC) and waist-to-hip ratio (WHR),and waist-toheight ratio (WHtR)–are employed as successor indicators of visceral obesity to predict morbidity and mortality at the population level [1]. BMI traces back to the pioneering 1800s statistician Quetelet, who postulated a power-law relationship between height and weight [2]. BMI, which is calculated using weight divided by the square of an individual’s height in meters (kg/m^2^), is considered to be the most useful population-level measure of obesity and is a simple index to classify underweight, overweight and obesity in adults. The WHO has classified overweight and obesity in adults based on various BMI cutoff points [3]. These BMI-based obesity guidelines have been accompanied by doubt as to the validity of BMI as an indicator of dangerous obesity. BMI does not identify between muscle and Fat reposition, and there is evidence that whereas higher Fat mass is associated with greater risk of premature death, higher muscle mass reduces risk [4]. Fatness has been the most widely used anthropometric measure and has a strong relationship with long-term cardio metabolic outcomes [5]. Nevertheless, there are some pitfalls in the results of BMI analysis. In other words, the relationship between BMI and body composition is affected by individual factors such as aging, physical activity, ethnicity, and gender [6].

The other standard obesity indices related to cardio metabolic risk factors are WC, body Fat percentage (BFP), waist-to-hip ratio (WHR), and waist-to-height ratio (WHtR) [7]. Obesity prevalence among adult diabetic patients in the region of our study—namely, Iran—is about 76% and its effect on diabetic complications has been confirmed [8, 9]. Obesity plays an important role in creating cardio metabolic complications, including hypertension, type 2 diabetes mellitus (T2DM), dyslipidemia, and certain cancers [10]. The exact definition of body Fat could provide clinically useful guidance for researchers and physicians to estimate disease risks in patients with obesity and optimize prophylactic or therapeutic treatments for such patients [11]. Besides, as BMI cannot distinguish between Fat and lean body mass, most obesity indices are only the markers of central obesity, such as neck circumference (NC), hip circumference (HC), WHR, WC, and BFP.

Research has shown that most of these indices have a strong relationship with BMI [12]. The BFP of human or other living beings is the total mass of Fat divided by total body mass. Previous studies have demonstrated that BFP can represent the body composition more exactly than BMI even through both BMI and BFP have been used for assessing human health risks, such as cardiovascular risks [13]. Research has revealed that BMI has a significantly positive correlation with BFP and with height [14].

Given the aforementioned description, in this study the application of BMI and BFP in order to identify the metabolic complications of patients with T2DM was evaluated. Thus, this study aimed to compare the performance of BMI and BFP in recognizing the main risk factors and new appearing risk factors for metabolic syndrome in diabetic patients of Yazd, Iran.

## Methods

### Study participants

The present cross-sectional, observational study was carried out from January 2016 to April 2017 in Yazd Diabetes Research Center, Iran. The census method was used in this study. A total of 1022 diabetic patients participated in this study. The data were collected using their health records. All participants had a history of T2DM and were verbally questioned about active cancers, chronic infectious diseases, untreated hypothyroidism, Cushing’s syndrome, and pheochromocytoma. Informed written consent forms were obtained from all participants, and the local medical ethics’ committee approved the study protocol.

### Anthropometric measurements and body composition analyses

Weight and height: Height (m) and weight (kg) were each measured in the standing position to calculate the body mass index BMI = body weight/height2 (kg/m^2^). Their WC was measured at the end of expiration at the midpoint of the iliac crest and the lowest rib [15]. The Body Fat percentage were also obtained by Dornberg formula. In this formula, the zero coefficients for women were given one for men in the gender category. The following equation is known as the Dornberg formula: BodyFat = (1/2*BMI) + (0/23*Age)-(10/8*Gender)-5/4 (2) [16]. Overweight was diagnosed if a participant had a BMI ≥25 kg/m^2^ (both male and female) or BFP ≥25% (male)/ ≥32%(female) according to Asian criteria [16].

### Biochemical measurements

Fasting blood samples were collected from the participants. All samples were then analyzed for total cholesterol, triglycerides, low-density lipoprotein-cholesterol (LDL-C), high-density lipoprotein-cholesterol (HDL-C), glucose, and hemoglobin A1C (HbA1c). The systolic blood pressure (SBP) and diastolic blood pressure (DBP) were measured on the right arm using an electronic sphygmomanometer [17].

### Statistical analyses

For normally distributed data, they were presented as means ± standard deviations; data were presented as median and range for not normally distributed data. Statistical analyses were carried out using SPSS version 16 for Windows (SPSS, Chicago, IL). Data were analyzed using t-test, Mann–Whitney U test, Chi-square test, and ANOVA. The correlation between BMI and BFP was calculated by Pearson’s correlation test. All participants were separated into 4 groups based on BMI and BFP cutoff points: normal weight and Non-Fat (Group A), BMI < 25.0 kg/m2 for women and men, and BFP < 32% for women and < 25% for men; overweight and Non-Fat (Group B), BMI ≥25.0 kg/m^2^ and BFP < 32% for women and < 25% for men; normal weight and Fat (Group C), BMI < 25.0 kg/m2 and BFP ≥ 32% for women and ≥ 25% for men; and overweight and Fat (Group D), BMI ≥25.0 kg/m2 and BFP ≥32% for women and ≥ 25% for men. An improved version of the International Diabetes Federation criteria (IDF) was used for identifying of the participant who have metabolic syndrome [10]. According to the IDF definition, metabolic syndrome is existing if at least 3 of the following criteria are met: WC > 102 cm (men) or > 88 cm (women), fasting glucose > 1 mmol/l, triglycerides < 1.5 mmol/l (150 mg/dl), high-density lipoprotein cholesterol (HDL-C) < 0.4 mmol/l(40 mg/dl) (men) or < 0.5 mmol/l(50 mg/dl) (women), blood pressure > 130/85 mmHg (13/8 CmHg), or treatment for previously recognized hypertension. To carry out a risk analysis among patient groups, the odds ratio of cardiovascular risk factors in groups 2, 3, and 4, compared to group 1 (used as a reference) was analyzed by a multivariate logistic regression model adjusted for sex, age, and group (divided into four groups: < 40, 40–< 50, 50–< 60, ≥60 years). A backward, stepwise multivariable logistic regression model adjusted for age, height, and weight and smoking was employed to specify the association of each metabolic syndrome risk (set as a dependent variable) with BMI and BFP based on grouping which was set as independent variables. Sex stratification was used in the analysis. A *p*-value of < 0.05 (two-tailed) was considered to be statistically significant.

## Results

A total of 1022 diabetic patients with the mean age of 55.3 ± 10.7 (499 males and 523 females) were eligible for the study and were classified as overweight/normal weight using BMI and Fat/Non-Fat using BFP cutoff points. Table 1 presents a summary of the demographic characteristics and biochemical test results for all participants.

**Table 1:** Characteristics of diabetic patients defined as Non-Fat and Fat using obesity measures

Variables	BMI			BFP		
	Normal weight (***N*** = 169)	Overweight (***N*** = 65)	***P***-value	Non-Fat (***N*** = 139)	Fat (***N*** = 95)	***P-***value
**Age**	**57 ± 11**	**55 ± 10**	**0.015**	**52 ± 11**	**56 ± 10**	**< 0.001**
**WC**	**90.31 ± 7.32**	**104.69 ± 9.44**	**0.000**	**96.2 ± 8.38**	**103.1 ± 11.05**	**< 0.001**
**BMI**	**22.7 ± 1.89**	**30.37 ± 4.08**	**0.000**	**26.59 ± 3.17**	**29.26 ± 5.21**	**< 0.001**
**BFP**	**31.75 ± 5.94**	**37.42 ± 6.73**	**0.000**	**27.84 ± 3.45**	**38.96 ± 5.45**	**< 0.001**
**Systolic blood pressure**	**12.84 ± 1.96**	**13.38 ± 1.98**	**0.000**	**12.95 ± 1.99**	**13.36 ± 1.97**	**0.004**
**diastolic**	**7.22 ± 1.12**	**7.4 ± 1.15**	**0.02**	**7.14 ± 1.14**	**7.4 ± 1.142**	**< 0.001**
**LDL**	**107.6 ± 40.47**	**98.26 ± 35.7**	**0.001**	**104.58 ± 37.17**	**99.02 ± 36.99**	**0.036**
**HDL**	**45.12 ± 14.51**	**43.2 ± 12.35**	**.044**	**45.7 ± 11.11**	**42.9 ± 13.41**	**0.003**
**triglyceride**	**150.67 ± 74.75**	**179.13 ± 76.04**	**0.000**	**176.89 ± 71.35**	**189.24 ± 66.92**	**0.002**
**fasting glucose**	**162.78 ± 66.27**	**182.23 ± 78.98**	**0.000**	**167.69 ± 67.9**	**178.19 ± 68.38**	**< 0.001**
**HbA1C**	**8.53 ± 2.15**	**7.9 ± 1.84**	**0.000**	**8.16 ± 2.01**	**8.08 ± 1.86**	**0.259**
**2hpp**	**237.35 ± 91.7**	**272.56 ± 80.1**	**0.000**	**238.3 ± 90.93**	**250.34 ± 80.41**	**0.01**

Notes: Data except for number (%) are the mean (standard error of mean). All biochemical markers are expressed in Système International units.

Abbreviations: *WC* Waist circumference, *BFP* Body Fat percentage, *BMI* body mass, *HDL* high density lipoprotein, *LDL* low density lipoprotein, *HbA1c* hemoglobin A1c, *2hpp* 2 hour Post Prandial. Denotes statistically signifcant diference between groups(*p*-value< 0.05)

In the first part of Table 1, the participants’ BMI indices were divided into two groups: normal weight and overweight. In the second part of the table, individuals were divided into two groups: Non-Fat and Fat, based on BFP. According to their BMI, 23.4 and 76.6% of the participants were classified as normal and overweight, respectively; however, based on BFP, 25.7 and 74.3% of the study population were identified as Non-Fat and Fat, respectively.

The results showed that the mean of age, WC, systolic blood pressure, diastolic blood pressure, LDL, HDL, triglyceride, fasting glucose, and 2hpp in two groups within T2DM patients with normal BFP were significantly lower than those with high BFP (*p*-value < 0.05). The mean difference of HbA1C among groups was not significant (*p*-value > 0.05). However, the mean of all factors except HDL and HBA1C in lean participants was higher than the fat population.

### Groups characteristics based on different obesity level

Participant diabetic patients were classified into four (A-D) groups based on whether they overpass BMI or BFP cut-off points (Table 2). As for WC, BMI, BFP index, blood pressure, diastolic, LDL, triglyceride, and fasting glucose, significant differences were observed among the parameter values for all four groups, which were clearly greater in the participants with overweight and Non-Fat and overweight and Fat (WC, BMI, Body Fat index, blood pressure, diastolic, LDL, triglyceride, fasting glucose in Group B and Group D were significantly higher than Group A and Group C). Accordingly, the results showed an increase in the prevalence of metabolic syndrome (more than 60%) in the overweight and/or FAT (Group D) compared with the normal weight and Non-Fat group (Group A)**.**

**Table 2:** Demographic characteristics and metabolic parameters of diabetic patients regarding classification by body mass index (BMI) and body Fat percentage (BFP)

Variables	Categories of obesity indices according to Percentage of body Fat				
	Group A (***N*** = 66)	Group B (***N*** = 197)	Group C (***N*** = 173)	Group D (***N*** = 586)	*P*-VALUE
**Age**	**57.5 ± 13.58**	**50.46 ± 9.97**	**56.61 ± 10.21**	**56.41 ± 10.35**	**0.000**
**Waist circumference**	**88.92 ± 7.55**	**98.64 ± 7.15**	**90.84 ± 7.19**	**106.7 ± 9.24**	**0.000**
**BMI**	**22.37 ± 2.36**	**28.00 ± 1.91**	**22.82 ± 1.67**	**31.16 ± 4.31**	**0.000**
**Body Fat index**	**24.37 ± 4.1**	**29.01 ± 2.2**	**34.57 ± 3.66**	**40.25 ± 5.21**	**0.000**
**Blood pressure**	**13.16 ± 2.25**	**12.87 ± 1.9**	**12.72 ± 1.83**	**13.55 ± 1.97**	**0.001**
**diastolic**	**6.98 ± 1.23**	**7.20 ± 1.10**	**7.31 ± 1.07**	**7.47 ± 1.15**	**0.001**
**LDL**	**96.61 ± 39.79**	**102.89 ± 36.2**	**106.88 ± 40.81**	**109.7 ± 35.48**	**0.003**
**HDL**	**48.42 ± 14.44**	**44.79 ± 9.62**	**43.87 ± 14.39**	**42.67 ± 13.10**	**0.000**
**Triglyceride**	**161.84 ± 63.18**	**187.74 ± 67.04**	**159.79 ± 66.62**	**181.93 ± 71.96**	**0.000**
**Fasting glucose**	**162.74 ± 64.88**	**171.66 ± 69.61**	**186.53 ± 81.02**	**177.12 ± 62.51**	**0.000**
**hba1c**	**8.45 ± 2.15**	**8.06 ± 1.96**	**8.56 ± 2.16**	**8.91 ± 1.74**	**0.000**
**2hpp**	**230.21 ± 95.59**	**232.24 ± 88.18**	**277.76 ± 98.23**	**262.87 ± 90.71**	**0.000**
**Metabolic syndrome, n (%)**	**28(42.42%)**	**64(36.99%)**	**132(67%)**	**408(69.62%)**	**0.000**

Notes: Data except for number (%) are the mean (standard error of mean). Individual group is defined as follows: normal weight and Non-Fat (Group A), BMI < 25.0 kg/m2 for women and men, and BFP < 32% for women and < 25% for men; overweight and Non-Fat (Group B), BMI ≥25.0 kg/m^2^ and BFP < 32% for women and < 25% for men; normal weight and Fat (Group C), BMI < 25.0 kg/m2 and BFP ≥ 32% for women and ≥ 25% for men; and overweight and FAT (Group D), BMI ≥25.0 kg/m2 and BFP ≥32% for women and ≥ 25% for men. According to the IDF definition, metabolic syndrome is existing if at least 3 of the following criteria are met: WC > 102 cm (men) or > 88 cm (women), fasting glucose > 1 mmol/l, triglycerides < 1.5 mmol/l (150 mg/dl), high-density lipoprotein cholesterol (HDL-C) < 0.4 mmol/l(40 mg/dl) (men) or < 0.5 mmol/l(50 mg/dl) (women), blood pressure > 130/85 mmHg (13/8 CmHg), or treatment for previously recognized hypertension. Metabolic syndrome is defined using the NCEP ATP III criteria modified for Asian population

Abbreviations: *W*C Waist circumference, *BFP* Body Fat percentage, *BMI* body mass, *HDL* high density lipoprotein, *LDL* low density lipoprotein, *HbA1c* hemoglobin A1c, *2hpp* 2 hour Post Prandial. Denotes statistically signifcant diference between groups (*p*-value< 0.05)

**Fig. 1:**
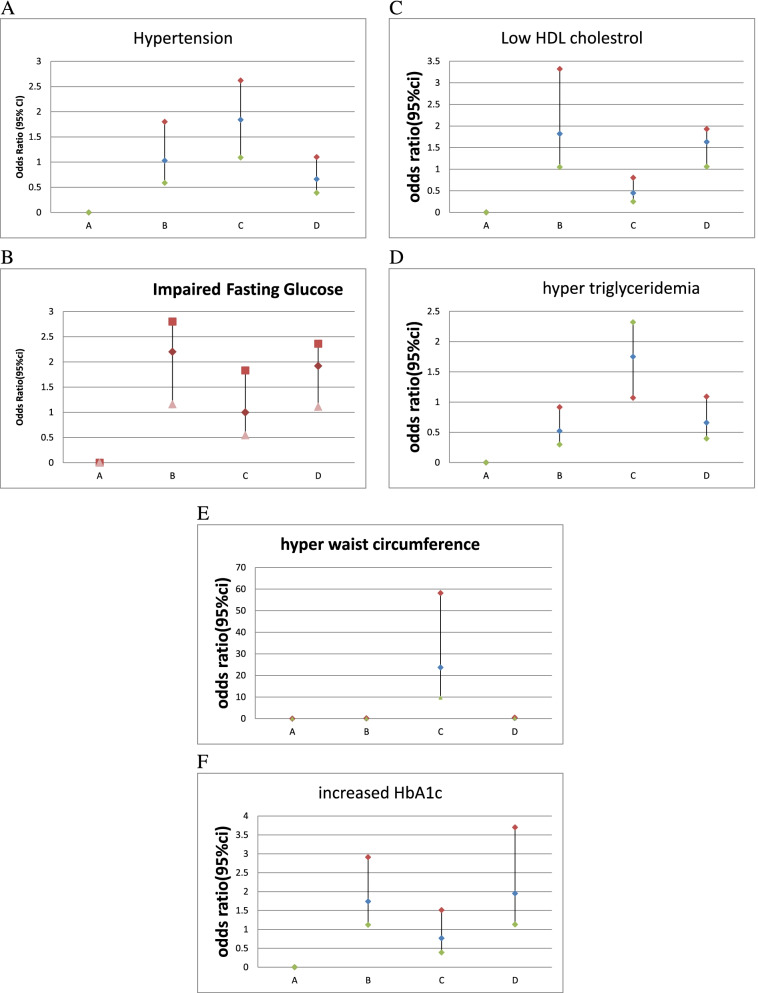
Multinomial logistic regression analysis for (**A**) hypertension, (**B**) impaired fasting glucose, (**C**) hypo HDL cholesterolemia, (**D**) hypertriglyceridemia, (**E**) hyper waist circumference, (**F**) increased HbA1c pattern for the following groups compared with the reference normal-weight and NON-FAT. group (Group **A**): overweight and NON-FAT (Group **B**), BMI ≥25.0 kg/m2 for women and ≥ 27.0 kg/m2 for men, and BFP < 35% for women and < 25% for men; normal weight and Fat (Group **C**), BMI (BMI) < 25.0 kg/m2 for women and < 27.0 kg/m2 for men, and Body Fat percentage (BFP) ≥35% for women and ≥ 25% for men; and overweight and Fat (Group D), BMI ≥25.0 kg/m2 for women and ≥ 27.0 kg/m2 for men, and BFP ≥35% for women and ≥ 25% for men

To compare cardio metabolic and metabolic syndrome risk factors between overweight and/or Fat and normal weight and Non-Fat groups, a multinomial logistic regression model was used (Fig. 1).

The odds ratios (95% confidence interval) for hypertension (Fig. 1A) were significant in group C (1.84 [1.09–2.62]), but they were not significant in Group B (1.03 [0.58–1.8]) and group D (0.66[0.39–1.1]). Fasting glucose (Fig. 1B) was statistically significant in the groups D and B. OR_S_ (95% CIs) were [1.92(1.11–2.33)] and [2.2(1.16–2.1)], for the groups D and B, respectively. Low HDL (Fig. 1C) was statistically significant in group B and group D: ORs (95% CIs) were 1.82 (1.05–1.97) and 1.63 (1.06–1.93), respectively. Moreover, hypertriglyceridemia was statistically significant only in group C (1.75 [1.07–2.32]) (Fig. 1D). The ORs (95% CIs) for increased HbA1c (Fig. 1E) were significant in group D (1.95 [1.13–3.7]) and group B (1.74 [1.12–2.91]), but they were not significant in group C (0.76 [0.38–1.51]). High WC was only statistically significant in group C (23.71[9.67–58.18]) (Fig. 1F).

### Correlations among BMI, BFP and metabolic syndrome risk factors classification by sex

A backward, stepwise removal regression was run to check the relationships among the metabolic syndrome risk factors associated with BMI and BFP. The variables were adjusted by age and height the analysis and are shown in Table 3. According to the conducted analysis, the following results were obtained: BMI was associated with hypertension in men (*p* < 0.05); elevated fasting glucose was associated with BFP in men (*p* < 0.05); high WC was associated with BMI in men and women (*p* < 0.05); low HDL was associated with BFP in women (*P* < 0.05) and hypertriglyceridemia was associated with BMI and BFP in men (*P* < 0.05).

**Table 3:** Logistic binary regression analysis of obesity measures associated with metabolic syndrome risk factors

Dependent variables	Independent variables	Men		Women	
		OR (95% CI)	*P*-value	OR (95% CI)	*P*-value
**Blood pressure**	**BMI**	**2.17(1.49–3.17)**	< 0.001	**–**	**–**
	**BFP**	**–**	**–**	**1.63(1.14–2.32)**	**0.007**
**Fasting glucose**	**BMI**	**–**	**–**	**2.23(1.11–4.91)**	**–**
	**BFP**	**4.68(0.4–54.19)**	< 0.001	**–**	**–**
**Waist circumference**	**BMI**	**60.93(23.99–154.75)**	< 0.001	**31.56(15.26–65.27)**	< 0.001
	**BFP**	**–**	**–**	**–**	**–**
**HDL**	**BMI**	**–**	**–**	**–**	**–**
	**BFP**	**–**	**–**	**3.23(0.843–4.05)**	**0.03**
**Triglyceride**	**BMI**	**2.39(1.48–3.18)**	< 0.001	**–**	**–**
	**BFP**	**–**	**–**	**27.34(25.01–45.87)**	**0.000**

Notes: a Data are from logistic regression analyses adjusted for age, sex; Dashes mean variable removed from the equation by backward stepwise selection. According to the IDF definition, metabolic syndrome is existing if at least 3 of the following criteria are met: WC > 102 cm (men) or > 88 cm (women), fasting glucose > 1 mmol/l, triglycerides < 1.5 mmol/l (150 mg/dl), high-density lipoprotein cholesterol (HDL-C) < 0.4 mmol/l(40 mg/dl) (men) or < 0.5 mmol/l(50 mg/dl) (women), blood pressure > 130/85 mmHg (13/8 CmHg), or treatment for previously recognized hypertension. Metabolic syndrome is defined using the NCEP ATP III criteria modified for Asian population

Abbreviations: *WC* Waist circumference, *BFP* Body Fat percentage, *BMI* Body mass, *HDL* High density lipoprotein, *LDL* Low density lipoprotein, *HbA1c* hemoglobin A1C, *2hpp* 2 hour Post Prandial. Denotes statistically significant difference between groups (*p*-value< 0.05)

## Discussion

BMI (body mass index) and BFP (body Fat percentage) are both clinically used to diagnose obesity. People in Asian countries have a higher body Fat percentage than other people, while their BMI is the same. The results of many studies have shown that gender has a significant relationship with Fatty tissues so that women have a higher percentage of Fat than men while their BMI is the same [18, 19].

The results of our study showed that there is a strong correlation between BMI and BFP in the whole diabetic patients and gender subgroups. This indicates that sex-specific cutoff points for BMI and BFP indices are appropriate for specifying additional body weight and body Fat for the Iranian diabetic patients. According to such criteria, 75.9% of the participants were identified to be overweight or obese using the BMI cutoff points, but 74.3% were specified to be Fat using the BFP cutoff points.

In a study by Vanavanan, carried out on a total of 234 outpatients with the minimum age of 20 years it was observed that 27.8% were obese based on BMI and 40.6% were Fat using the BFP cutoff points [20]. In this study, 173 (17%) individuals with normal weight (BMI < 25 kg / m2) had high BFP. Also, 195 participants (19.2%) with normal BFP had a highly excessive BMI. In Group B (overweight and Non-Fat) and Group D (overweight and Fat), there was a significant increase in the WC (waist circumference), and triglyceride compared with Group A (normal weight and Non-Fat) and Group C (normal weight and Fat). However, there was a significant increase in the blood pressure, diastolic, HDL, fasting glucose, HbA1c, 2hpp in Group C (normal weight and Fat) and Group D (overweight and Fat) as compared to Group A (normal weight and Non-Fat) and B (overweight and Non-Fat).

These results also indicate the strong diagnostic function of BMI in measuring increased body Fat in Iranian diabetic patients. In another study by Romero-Corral, despite a good correlation between BMI and BFP in a large sample of adults in the US population, the diagnostic accuracy of BMI to diagnose obesity was found to be limited, particularly in individuals in the intermediate BMI ranges. The direct but simple measures of body Fatness and measures of body Fat distribution may be helpful in such individuals to further stratify them according to their level of body Fatness [21]. Moreover, a large difference was observed in the abilities of BMI and BFP indices to detect and predict metabolic syndrome risk factors, as shown by ORs (Table 3). The association between BMI and hypertension in men was significant, and the odds ratio in men with high BMI was 2.17 times higher than those with normal BMI. In women, the relationship between BMI and hypertension was not significant. For men, BMI can be a better predictor of hypertension than BFP. The association of BFP with hypertension in women was significant; moreover, the odds ratio in women with high BFP was 1.63 times higher than those with low BFP. As a result, for women, BFP can be regarded as a better predictor of hypertension, but in men, body Fat cannot be a good predictor of hypertension. Likewise, a study conducted by Hou in China concluded that BMI could reflect body mass, and this was associated with blood viscosity and blood pressure [22].

Although the relationship between BMI and hyperglycemia was not significant in men, it was significant in women. The odds ratio in women with high BMI was 2.32 times higher than women with normal BMI. Thus, BMI of woman can be a better predictor of BFP than hyperglycemia. The relationship between BFP and hyperglycemia in men was significant and the odds ratio in men with high FBP was 4.68 times higher than men with low BFP. Although BFP is a better predictor of hyperglycemia in men, but BFP it was not a good predictor of hyperglycemia in women. According to the results of Vanavanan’s study, the glucose metabolic profile of people with BMI and high BFP can cause an increased risk of metabolic syndrome [20]. In men and women, the relationship between BMI and hypo HDL cholesterolemia was not significant. In women, the relationship between BFP and hypo HDL cholesterolemia was significant and the odds ratio for women with high BFP was 3.23 times higher than those with low BFP. According to the results of this study, BMI was associated with hyper WC in both sexes. The odds ratio in men with high BMI was 60.93 times higher than those with normal BMI, and odds ratio in women with high BMI was 31.56 times higher than those with normal BMI. Thus, BMI can be considered as a better predictor of high WC in comparison with BFP in both men and women. In women and men, the relationship between BFP and high hyper WC was not significant. Therefore, BFP cannot be regarded as a good predictor of WC. BMI was a better predictor of high WC in comparison with BFP in both men and women.

The relationship between BMI and hypertriglyceridemia in men was significant, and the odds ratio in men with high BMI was 2.39 times higher than those with normal BMI. In contrast, the relationship between BMI and hypertriglyceridemia was not significant in women. Thus, BMI was a better predictor of hypertriglyceridemia than BFP in men. The association of BFP with hypertriglyceridemia in women was significant, and the odds ratio for women with high BFP was 27.34 times higher than those with low BFP. As a result, BFP can be a better predictor of hypertriglyceridemia in women; in men, however, body Fat was not a good predictor of the hypertriglyceridemia. In another study by Blaak, there were significant relationships among fasting glucose, triglycerides, and BFP in men, but in women, this relationship was not significant. Hence, in women, BMI is a good predictor of risk factors for metabolic syndrome. Women have a higher Fat percentage than men because of differences in Fatty acid and oxidation. Moreover, women tend to store more Fat in the gluteal–femoral region whereas men store more Fat in the visceral storeroom [23]. The strength of our study was based on a database of good number of diabetic patients. The limitation of our study was that we measured the PBF using the Dornberg equation, which may not be very accurate. Our data was limited to the city of Yazd, Therefore, further studies involving larger and different Iranian populations are required to confirm the findings presented herein.

## Conclusion

This study was conducted on diabetic patients of Iran. Significant difference was observed between BMI and BFP to predictability of metabolic syndrome risk factors across sexes. For men, BMI can be a better predictor of hypertension than BFP. In women, BFP can be regarded as a better predictor of hypertension. For women, BMI can be a better predictor of hyperglycemia than BFP. In men, BFP is a better predictor of hyperglycemia, while BFP was not a good predictor of hyperglycemia in women. In men and women, the relationship between BMI and hypo HDL cholesterolemia was not significant. In women, the relationship between BFP and hypo HDL cholesterolemia was significant. Thus, BFP was a better predictor of hypo HDL than BMI in women. According to the results of this study, BMI can be considered as a better predictor of high WC in comparison with BFP in both men and women. BMI was a better predictor of hypertriglyceridemia than BFP in men. BFP can be a better predictor of hypertriglyceridemia in women; in men, however, body Fat was not a good predictor of the hypertriglyceridemia. No single measure may provide a comprehensive risk predication as shown in this study. Therefore, BMI and BFP should be considered in at risk population at the screening activities.

## Data Availability

The datasets used and/or analyzed during the current study available from the corresponding author on reasonable request.

## Abbreviations

BMI: Body mass index
BFP: Percentage of body Fat
